# A guide for the utilization of Health Insurance Review and Assessment Service National Patient Samples

**DOI:** 10.4178/epih/e2014008

**Published:** 2014-07-30

**Authors:** Logyoung Kim, Jee-Ae Kim, Sanghyun Kim

**Affiliations:** Health Insurance Review and Assessment Service, Seoul, Korea

**Keywords:** National Health Insurance, Claims data, Stratified sampling, Healthcare services, Cross-sectional data

## Abstract

The claims data of the Health Insurance Review and Assessment Service (HIRA) is an important source of information for healthcare service research. The claims data of HIRA is collected when healthcare service providers submit a claim to HIRA to be reimbursed for a service that they provided to patients. To improve the accessibility of healthcare service researchers to claims data of HIRA, HIRA has developed the Patient Samples which are extracted using a stratified randomized sampling method. The Patient Samples of HIRA consist of five tables: a table for general information (Table 20) containing socio-demographic information such as gender, age and medical aid, indicators for inpatient and outpatient services; a table for specific information on healthcare services provided (Table 30); a table for diagnostic information (Table 40); a table for outpatient prescriptions (Table 53) and a table for information on healthcare service providers (Table of providers). Researchers who are interested in using the Patient Sample data for research can apply via HIRA’s website (https://www.hira.or.kr).

## INTRODUCTION

South Korea has a universal health coverage system that the National Health Insurance covers approximately 98% of the overall Korean population. The claims data of Health Insurance Review and Assessment Service (HIRA) contains 46 million patients per year that account for 90% of the total population in Korea and include claims from almost 80,000 healthcare service providers across South Korea as of 2011. The claims data of HIRA includes patients’ diagnosis, treatment, procedures, surgical history, and prescription drugs which provide a valuable resource for healthcare service research. However, the complex structure and vast volume of claims data require considerable efforts on the part of the researcher to understand them. In addition, the applications to use the claims data of HIRA and their deliberation process take significant amount of time, and these processes restrict the researcher from accessing the data. Furthermore, the vast volume of the data can result in their inefficiency in conducting research. To resolve these limitations and improve accessibility for researchers to use the claims data, HIRA has developed the Patient Sample data that had passed validity tests performed by five different institutions.

The Patient Samples are stratified random samples of claims data of HIRA. The sizes for the samples had carefully been calculated and extracted to improve representativeness of the socio-demographic characteristics, diagnosis, healthcare services including prescription drugs for Korean patients on a year basis. HIRA has provided four samples with different groups in order to enhance reliability and representativeness by extracting a sample from a specific area. As claims data of HIRA consist of 10% of inpatients and 90% of outpatients, the National Patient Sample (NPS) may not have enough cases to investigate inpatient services for severe health conditions. To support the research in groups of which representativeness is not ensured in NPS data, currently available are samples that were separately sample: National Patient Sample (HIRA-NPS), National Inpatient Sample (HIRA-NIS), Adult Patient Sample (HIRA-APS), and Pediatric Patient Sample (HIRA-PPS) (Table 1).

The Patient Samples are annually updated each year because they are developed through the accumulation of claims data over a year-long cycle. However, the Patient Samples are cross-sectional and different patients are selected for the sample data each year to protect their privacy. As such, a specific individual or healthcare service provider is not subject to a longitudinal data extraction, even within the same type of Patient Samples. Therefore, it is not possible to conduct research that requires a long-term following of patients with the Patient Samples.

### Case study from abroad

In the US, a federal research institution called the Agency for Healthcare Research and Quality (AHRQ) performs and supports research associated with healthcare services. AHRQ collects data from 37 state governments, community, and the healthcare industry organizations to form a healthcare database. One of AHRQ’s research programs, Healthcare Cost and Utilization Project (HCUP), constitutes the largest healthcare database in the US. Among the data provided by HCUP, the National Inpatient Sample (NIS) is the most comprehensive inpatient data, which includes all the healthcare communities within the American Hospital Association, except for physical therapy institutions. The NIS is based on data collected from 3,900 member healthcare institutions spanning 37 states. Among the member healthcare institutions, approximately 20% (800-1,100 institutions) are extracted for sampling to include all of the inpatient data (5-8 million cases) from the sample institutions (Table 2).

### Data resource area and population coverage

The claims data of HIRA is collected when healthcare service providers in South Korea seek reimbursements for healthcare services that the National Health Insurance Corporation agrees to cover. The annual number of Korean patients that submitted health insurance claims is approximately 46 million. The claims data of HIRA is a national data compiled from healthcare providers across the country that corresponds to the number of claims submitted by patients. In addition, the claims from patients with medical aid program, government expenditures, and veteran patients are also included in the claims data.

## MEASURES

### Extraction method

Because the standards of differentiation for claims submitted to HIRA are clearly defined, the Patient Samples adopted stratified sampling, a probabilistic sample extraction method. Based on the two stratum of sex (2 strata) and age (16 strata), the sample was divided into a total of 32 strata before random extraction. Demographically stratification of claims data at the patient level secures representativeness of the claims data in accounting for the time series of data which differ based on types of the healthcare service settings (inpatient or outpatient), the cycle of claims data submissions from providers (daily or monthly), and types of diseases.

As the charge in the National Health Insurance claims data exhibit the maximum variance and best reflect the characteristics of the claims data, the charge was chosen as a sample variable. Under the assumption of acceptable sampling error range and normal distribution, the standard deviation and the sample size were calculated with the following equation.

Using the above equation, the patient sample sizes that best reflects the representativeness of the overall claims data was determined as shown in the Table 3. Table 3 presents the comparison result between the estimated population derived from adding weighted values to the HIRA sample data and the actual population. The estimated population and the actual population exhibited a 95% concordance demonstrating a high level of representativeness.

### Explanation of variables

The Patient Samples consist of five tables: Table 20 (general specifications), Table 30 (health services), Table 40 (diagnosis information), Table 53 (outpatient prescription), and Table of Providers (Healthcare Service Provider Information).

All tables are linkable using a key ID. Table 20 (general specifications) includes the general characteristics of the patient such as socio-demographic characteristics (gender, age, and medical-aid program), major diagnosis, secondary diagnosis, payer’s amount, and patient’s out-of-pocket cost. Table 30 (health services) includes details in inpatient and outpatient healthcare services provided to patients such as procedures, treatment and prescription drugs for inpatients. Table 40 (diagnosis information) contains all of the diagnosis information that patients have had. Table 40 is used when a patient’s concomitant disease or a history of all conditions is deemed necessary. Table 53 (outpatient prescriptions) shows drug information that had been prescribed recorded for outpatients such as active ingredients, dosage and days of supply. Finally, Table of Providers includes information about the healthcare service provider that the patient had visited, including the type of healthcare service provider (primary care, secondary care, tertiary care), location, sizes, and ownership type (Table 4).

### Strengths and weaknesses

The Patient Samples have several important strengths. The first one is representativeness of the total patient population in South Korea and has advantages in generalization for the population. Secondly, the Patient Samples have comprehensive yet specific information on healthcare services including prescription drugs provided under the fee-for-service system. Thirdly, as the Patient Samples passed the validity test [1], they are proved efficient in estimating population to save costs and time in conducting research. HIRA has made memorandum of understanding (MOU) with five academic societies in healthcare service (Korean Society for Preventive Medicine, Korean Association of Health Economics and Policy, Korean Society of Health Information and Health Statistics, Korean Academy of Health Policy and Management, Korean Society of Epidemiology) and performed tests to validate with the Patient Samples.

Among the major test results, “Korean prevalence of diabetes and evaluations of dipeptidyl peptidase-4 inhibitor use” demonstrated that the estimate on diabetes prevalence using sample data aligned with that of population-analysis. Moreover, the estimate on prescription of hypoglycemic agents aligned with the results of population-analysis. Additionally, outpatient prescription rates of each hypoglycemic agent were all within the 95% confidence interval. In the test of “The burden of social cost estimates of diseases associated with vision loss and blindness,” the Patient Samples and the population both exhibited higher health service use in female patients with major eye diseases (cataracts, glaucoma, macular degeneration, diabetic changes in the retina, and retinal vein occlusion) than in male patients. Furthermore, the Patient Samples and the population exhibited similar age trends with respect to health service use in cases of cataracts, glaucoma, and macular degeneration.

Despite strengths described above, a few limitations need to be noted when researcher is interested in utilizing the Patient Samples for research. First, the accuracy of diagnosis has been an issue due to the nature of claims data which is collected with a purpose of reimbursing healthcare services not of clinical purpose. Hence, it is possible that diagnosis information in claims data is susceptible to up-coding by providers seeking for higher reimbursement rate or diagnosis remains in the data even when they are ruled out after running lab tests. This implies patients with a certain diagnosis would not necessarily mean that they have the disease corresponding to the diagnosis. The inaccuracy of diagnosis information in claims data may not be a problem only for the claims data of HIRA but also most other claims data although the problem with diagnosis information in the claims data of HIRA can be more serious due to the fee-for-service system and reimbursement policies.

The study shows that diagnosis in the claims data of HIRA tends to be more accurate in cases of severe diseases rather than frequently occurring mild diseases [2]. In addition, they exhibit greater accuracy in inpatient setting than outpatient cases, and in hospitals rather than clinics [2]. To address the inaccuracy of diagnosis information, researchers use operational definition to identify patients with a certain disease rather than simply using diagnosis in the data [3-5]. Secondly, the Sample data may not have sufficient cases for rare disease and a certain age group with lower frequency. Third, the socioeconomic characteristics and risk-factors such as patient’s income, education, location, weight, height, mortality, and health behaviors such as smoking, drinking, exercise amount are deficient, resulting in limitations in conducting through research. HIRA has projects to link data from other institutions to enrich the patient socioeconomic and risk-factor variables. Finally, the Patient Samples are cross-sectional, making a longitudinal study following a same individual patient over years impossible. To protect the privacy of a patient, a patient ID and the healthcare service provider ID, alternative IDs are given alternative ID so that patients and providers are not identifiable. Sensitive information such as rare diseases and legally designated infectious diseases that can lead to the identification of a patient was also removed from the from the data.

## DATA ACCESSIBILITY

Patient Samples can be obtained via website of HIRA by filling out the End User Agreement of the Patient Samples. The Patient Samples are provided in a DVD (text file) format and a fee for the samples is subject to be charged.

Go to HIRA website → government 3.0 information open → application for the use of medical information → sample data

http://www.hira.or.kr/dummy.do?pgmid=HIRAA070001000312&cmsurl=/cms/open/02/01/02/index.html.

Tel: 02-2182-2601

E-mail: kshyun84@hiramail.net

## AVAILABILITY OF DATA DICTIONARY IN ENGLISH

HIRA is currently working on the data dictionary in English.

## Figures and Tables

**Table 1.** Sample sizes and computation of each sample data

Sample type	Computation standard
HIRA - National Inpatient Sample (2009-2011)	700,000 inpatients per year (13%), approximately 400,000 outpatients per year (1%)
HIRA - National Patient Sample (2010-2011)	1.4 million patients overall per year (3%)
HIRA - Adult Patient Sample (2010-2011)	Approximately 1 million patients over the age of 65 per year (20%)
HIRA - Pediatric Patient Sample (2010-2011)	Approximately 1.1 million patients under the age of 20 per year (10%)

The size of each sample data is either 1.5 million patients or 20% of the total number in the corresponding area of the original data

HIRA, Health Insurance Review and Assessment Service.

**Table 2.** Comparison of the nations’ sample datasets

Country-based comparison	Korea (HIRA)	US (AHRQ)	Taiwan (NHIRD)
Sampling unit	Patient sampling	Hospital sampling	Patient sampling
Unit of data provided	Patient based	Institution case-based (discharge data)	Patient data
Stratification variable	Demographic characteristic (sex, age group)	Hospital characteristics Geographic location	Simple random sampling
Data providee	All researchers	All researchers	National research institutions and researchers (general public uses the educational data set)
Sampling unit	Inpatients, all patients, pediatric and teen patients, elderly patients under 1.5 million people per category	Approximately 7 million institutional cases (inpatient data)	Health-plan registrees, 1 million people

HIRA, Health Insurance Review and Assessment Service; AHRQ, Agency for Healthcare Research and Quality; NHIRD, National Health Insurance Research Database.

**Table 3.** Comparison between Patient Samples and the actual population (unit: person)

Sample type	Sample size (%)	Estimated population	Actual population
HIRA - National Patient Sample	1,375,842 (3)	45,861,321	47,026,505
HIRA - National Inpatient Sample	765,564 (13)	5,888,921	6,026,063
HIRA - Adult Patient Sample	1,073,183 (19)	5,365,917	5,650,511
HIRA - Pediatric Patient Sample	1,026,648 (10)	10,266,474	10,681,503

The percentage of the total patients in claims data.

Based on data from 2011.

**Table 4.** Main variables in the Patient Samples

Table	Variables
Table 20 (general specification)	Billing statement identification code (key ID), patient ID, provider’s ID, stratification variables, strata, age, gender, sample weight, DRG billing number, claims types, date of admission, insurance type, hospital arrival path way, major diagnosis, secondary diagnosis, injury from public service, days of care, initial date of care, final date of care, days in hospitalization, payer’s amount, patient’s out of pocket cost, total amount, surgical status
Table 30 (healthcare services)	Billing statement identification code (Key ID), service category, classification type, unit price, total price, daily dosages, days of supply, quantity of supply, service codes, drug codes
Table 40 (diagnosis information)	Billing statement identification code (key ID), indicator for major diagnosis, department, diagnosis
Table 53 (outpatient prescriptions)	Billing statement identification code (key ID), classification type, unit price, total price, daily dosages, days of supply, quantity of supply, service codes, drug codes
Table of Providers	Provider ID, type of providers, presence of special equipment (CT, MRI, PET), location, number of beds, number of staff per 50 beds - physicians, dentists, acupuncturists, and nurses

IDs are given an alternative ID to protect private information.
