# Supplementary material for: A guide for the utilization of Health Insurance Review and Assessment Service National Patient Samples
Source: Epidemiol Health. 2014 Jul 30;36:e2014008. doi: 10.4178/epih/e2014008 (PMC4151963; doi:10.4178/epih/e2014008)
Supplement: Supplementary file 1 [file epih-36-e2014008-supplemetal data.pdf]

A Guide to the Use of National Patient Samples

Logyoung Kim , Jee-Ae Kim, Sanghyun Kim

(Health Insurance Review and Assessment Service)

Logyoung Kim,

Health Insurance Review and Assessment Service

HIRA 1st Annext Seocho Peace Bldg. 11F, 22 Banpodaero, Seocho-gu

Seoul, Korea,137-927

tel. 02-2182-2515/ Fax. 02-6710-5834

E-mail: kimlog2@hiramail.net

Jeeae Kim ,

Health Insurance Review and Assessment Service

HIRA 1st Annext Seocho Peace Bldg. 11F, 22 Banpodaero, Seocho-gu

Seoul, Korea,137-927

tel. 02-2182-2600/ Fax. 02-6710-5836

E-mail: kja0813@hiramail.net

Sanghyun Kim

Health Insurance Review and Assessment Service

HIRA 1st Annext Seocho Peace Bldg. 11F, 22 Banpodaero, Seocho-gu

Seoul, Korea,137-927

tel. 02-2182-2601/ Fax. 02-6710-5834

E-mail: shhyun84@hiramail.net

Abstract

Claims data of Health Insurance Review and Assessment Service (HIRA) are an importance source of information for healthcare service research. Claims data are generated when healthcare service providers submit a claim to HIRA for review for reimbursement. In order to improve accessibility to HIRA data for healthcare service researcher, HIRA have developed 3% of national patient sample

data (HIRA-NPS) which are extracted using a stratified randomized sampling method. HIRA-NPS data consist of five tables: a table for general information (table 20) containing demographic information such as gender and age, indicators for inpatient and outpatient services, a table for specific information on services provided (table 30), a table for diagnostic information (table 40) and a table for outpatient prescriptions (table 53). Researchers who are interested in using HIRA-NPS for research can apply via HIRA's website (https://www.hira.or.kr).

Key Messages

HIRA-NPS 는

- 대한민국 전국민을 대상으로 전국의 요양기관으로부터 청구되는 건에 대한 청구자료로부터 추출
- Stratified random sampling 을 이용하여 전체 환자에 대하여 영역별(전체, 입원, 소아▪청소년, 노인)로 150 만 이내로 추출
- 환자의 1 년 동안의 진료내역, 시술 및 처치내역, 상병정보, 처방내역이 포함
- 년도별로 상이한 환자군이 추출된 cross sectional data

Introduction

환자표본자료란 방대한 양의 국민건강보험 청구자료를 바탕으로 범용성과 대표성을 갖도록 환자표본크기를 계산하여 환자의 1 년 동안 진료, 처치, 처방내역을 추출한 청구명세서의 축소판을 말한다.

건강보험 청구데이터는 요양기관이 의료서비스를 제고한 후 환자의 진료비용중 국민건강보험이 부담하는 부분에 대해 지급을 요청하기 위하여 건강보험심사평가원(심평원)에 급여 청구를 하면서 발생하는 자료이다.

한국의 국민건강보험 가입자는 전 인구의 98%를 커버하고 있고, 1 년간 건강보험 청구 환자수는 주민등록 인구의 90%에 해당하는 약 4 천 6 백만 명으로, 전국의 8 만여 개 요양기관으로부터의 청구 건이 포함되어 있다 (2011 년도 기준).

이러한 청구데이터는 환자의 진단명, 진료 및 시술 내역, 처방전약 등과 같은 상세하고 방대한 정보를 포함하고 있어 health-rlated research 에 매우 유용한 자료 이지만, 청구데이터는 방대한 양과 복잡한 구조로 인해, 연구자가 데이터를 이해하는데 상당한 노력을 요구하게 되며. 또한 청구데이터를 사용하기 위한 신청과 심의절차에도 일정 시간이 소요 되어 시의 적절한 활용에 제한이 있다. 또한 데이터의 방대한 양은

데이터를 처리하는데에 비효율성을 초래하기도 한다. 이러한 제한을 해결하고 연구자들의 보다 용이한 데이터 이용을 위하여 심평원은 5 개 학회의 타당도 검증을 거친 환자표본 데이터를 개발하여 제공하고 있다.

현재 건강보험심사평가원은 전체환자표본 자료에 이어, 입원환자표본자료(HIRA-NIS) 고령환자표본자료(HIRA-APS), 소아·청소년환자표본자료(HIRA-PPS)를 추가 개발하여 총 4 가지 종류의 환자표본자료를 제공 중이다 (표 1).

표 1. 환자표본자료 종류 및 산출 기준

표본자료 종류	산출 기준
HIRA-NIS (2009-2011)	1 년 단위 입원환자 약 70 만 명(13%), 외래환자 약 40 만 명(1%)
HIRA-NPS (2010-2011)	1 년 단위 전체환자 약 140 만 명(3%)
HIRA-APS (2010-2011)	1 년 단위 65 세 이상 환자 약 100 만 명(20%)
HIRA-PPS (2010-2011)	1 년 단위 20 세 미만 환자 약 110 만 명(10%)

※ 각 환자표본자료의 표본 한계치는 환자 수 150 만 명 또는 영역별 20%이내를 기준으로 함.

환자표본자료는 수시로 누적되는 청구자료의 1 년 동안의 데이터를 취합하여 개발되므로 1 년을 주기로 업데이트가 된다. 단 환자표본자료는 cross-sectional data 로 동일 종류 표본자료라고 할지라도 개인정보보호를 위하여 연도간에 환자 및 요양기관이 연속적으로 이루어지지 않고 년도마다 다른 환자들이 추출된다. 따라서 환자표본데이터의 환자들을 년도별로 연계는 불가능하다. 환자들의 장기추적이나 연계가 필요한 경우의 연구시에는 환자표본데이터의 사용은 제한이 있다.

외국의 사례

미국의 Agency for Healthcare Research and Quality(AHRQ)는 연방정부에 속한 연구기관으로 보건의료 서비스 분야에 관련된 연구를 수행, 지원한다. AHRQ 는 37 개 주정부 및 지역사회,

보건의료 산업체들로부터 데이터를 수집하여 의료 데이터베이스를 구축하고 있다. AHRQ 의 조사 프로그램 중 하나인 Healthcare Cost and Utilization Project(HCUP)는 미국에서 가장 큰 보건의료 데이터베이스를 구축하고 있고, HCUP 의 제공 자료 중 가장 포괄적인 전국 입원환자 표본자료(National Inpatient Sample, NIS)는 재활의료기관을 제외한 미국병원협회(American Hospital Association)에 속해있는 모든 의료 커뮤니티를 포함한다. NIS 는 커뮤니티에 가입된 37 개 주의 약 3,900 개의 의료기관으로 부터 수집된 데이터를 기반으로 하고 있으며, 가입 의료기관 중 매년 약 20%(800~1,100 개 기관)를 표본추출하여 추출된 의료기관의 전체 입원 자료(약 5 백만~8 백만 입원 건)를 포함하고 있다.

Table 2. Comparison of the nations sample dataset

국가별비교	건강보험심사평가원(HIRA)	미국(AHRQ)	대만(NHIRD)
**추출 단위**	-환자 추출	-병원 추출	-환자 추출
**제공 단위**	-환자 단위	-기관 에피소드 중심 (퇴원자료)	-환자 단위
**층화 변수**	-인구학적 특징(성, 연령구간)	-병원 특징 -지리적 위치	-단순무작위추출
**제공 대상**	-모든 연구자	-모든 연구자	-국가 연구기관 및 연구자 (일반인은 학습용 데이터셋 이용)
**표본 단위**	-입원환자, 전체환자, 소아·청소년환자, 노인환자 등 분야별로 150 만명 이내	-약 700 만 건 정도의 기관 에피소드(입원자료)	-건강보험 등록자 100 만명

우리나라의 건강보험심사평가원의 경우 환자표본자료를 다양화하여, 특정영역의 표본을 따로 추출함으로써 해당영역에 대한 자료의 타당도 및 대표성을 높였다. 건강보험 청구자료 중에서 입원환자가 차지하는 비중은 약 10%이고 외래환자는 약 90%를 차지하고 있기 때문에, 전체 환자표본자료를 가지고 중증질환과 같은 입원진료를 연구하기에는 대표성이 떨어지게 된다. 따라서 입원환자표본, 노인환자표본과 같이 특정영역에 대해 별도의 표본을 추출해야 할 필요성이 있으며 자료의 활용도도 높아질 수 있다.

Data resource area and population coverage

국민건강보험 청구자료는 우리나라의 의료기관에서 환자의 진료비용 중 국민건강보험이 부담하는 부분에 대해 지급의뢰를 하기 위해 건강보험심사평가원에 청구하는 자료이다. 우리나라의 1 년간 건강보험 청구 환자수는 약 4 천 6 백만 명이며, 이에 상응하여 건강보험 청구자료는 전국의 요양기관으로부터의 청구 건이 수시로 누적되는 전 국민 데이터이며, 의료급여, 국비, 보훈환자들의 청구자료도 포함한다.

Measures

1) 추출방법

건강보험심사평가원에 청구되는 청구자료는 층을 나눌 수 있는 기준이 명확하여 확률적 표본추출방법 중에 하나인 층화추출법을 택하였다.

인구학적 특성인 성별(2 개 구간)과 연령(16 개 구간) 2 개의 층화추출변수를 기준으로 총 32 개의 층으로 나누어 추출하였다. 입원·외래 또는 요양기관의 종별 구분에 따라 일자별 혹은 월별 분리 청구되는 병원 청구자료의 특성과 질병마다의 기준으로 에피소드를 갖는 임상자료의 시계열 측면을 감안하여, 인구학적으로 층화 후 환자 단위 표본 추출하는 방식이 가장 부합한 추출 방법으로 판단되었다.

국민건강보험 청구자료에서 청구금액은 최대분산을 가지며, 자료의 특징을 가장 잘 반영하므로 표본변수로 택하였다. 청구금액의 표본오차와 정규분포를 따른다는 가정 하에 표본편차를 계산하여 환자표본크기를 아래의 식을 통해 산출하였다.

$$n = \frac{(Z_{\frac{\alpha}{2}})^2 \sigma^2}{B^2} \quad (\sigma : \text{표준오차},\ B : \text{표본오차})$$

위의 식을 통하여 전체환자표본자료의 대표성을 확보할 수 있는 환자표본크기는 약 137 만 명으로, 전체 모집단에서 3%의 추출 비율을 산출하였다.

Table 3. Comparison between Patient sample data and the actual population

(unit: person)

Category		Patient Sample data	Estimated Population	Actual Population
HIRA-NPS	Total	1,375,842	45,861,321	47,026,505
	Male	665,423	22,180,719	22,839,915
	Female	710,419	23,680,602	24,186,590
HIRA-NIS	Total	765,564	5,888,921	6,026,063
	Male	339,491	2,611,455	2,689,847
	Female	426,073	3,277,466	3,336,216
HIRA-APS	Total	1,073,183	5,365,917	5,650,511
	Male	434,540	2,172,702	2,305,088
	Female	638,643	3,193,215	3,345,423
HIRA-PPS	Total	1,026,648	10,266,474	10,681,503
	Male	531,318	5,313,172	5,554,180
	Female	495,330	4,953,302	5,127,323

※ Based on data from 2011
※ Actual population was computed only in cases of general claims and separated claims.

<표 2>는 전체환자표본자료를 통해 가중치를 부여하여 실제 모집단 환자 수와 비교한 결과를 보여준다. 모집단 추정 결과와 실제 모집단을 비교한 결과, 약 97%의 일치율을 보여 높은 대표성이 나타남을 보였다.

2) 변수설명

전체환자표본자료는 기본적으로 5 가지 영역의 테이블로 구성되어 있다: Table20(명세서일반내역), Table30(진료내역), Table40(상병내역), Table53(원외처방전내역) 요양기관 Table(요양기관정보)

각 테이블들은 Key 변수 서로 연결이 가능하다. Table20(명세서일반내역)에는 수진자의 일반적인 특성이 포함되어 있으며, 인구학적 변수(성별, 연령)와 주상병(영어로), 부상병(영어로), 요양급여비용(영어로), 본인부담금(영어로) 등을 포함한다. Table30(진료내역)은 환자들이 외래 또는 입원 시 발생하는 진료행위와 원내처방이 된 약제정보(주성분코드(영어로))등에 대한 정보를 포함하고 있다.

Table40(상병내역)은 환자들의 모든 진단명 정보를 담고 있다. 환자의 동반상병 혹은 보유하고 있는 모든 질병 정보가 필요한 경우에 사용되는 테이블이다. 마지막으로 Table53(원외처방상세내역)은 원외처방(영어로)으로 이루어지는 모든 약제에 대한 정보를 보여주며, 마찬가지로 주성분코드로 제공되고 있다. 마지막으로 요양기관테이블은 환자가 진료를 받은 요양기관의 종별(영어), 지역(영어), 설립구분(영어) 등 요양기관의 정보를 포함하고 있다.

표 2. 전체환자표본자료 제공변수

전체환자표본자료 Table	포함 변수
Table20	명세서연결코드, 수진자대체키, 요양기관대체키, 층화변수, 서식코드, 연령, 성별, 가중치, 진료결과구분코드, 진료과목코드, 청구DRG번호, 청구구분코드, 청구형태코드, 최초입원일자, 보험자코드, 입원도착경로구분코드, 주상병, 부상병, 공상구분, 요양일수, 내원일수, 요양개시일자, 요양만료일자, 보험자부담금, 본인부담금, 요양급여비용총액, 수술여부, 특정기호구분코드
Table30	명세서연결코드, 항목코드, 분류유형코드, 분류코드, 가산적용여부코드, 단가, 일일투여량 또는 실시횟수, 총투여일수 또는 실시횟수, 금액, 일반명코드
Table40	명세서연결코드, 일련번호, 진료과목코드, 상병코드
Table53	명세서연결코드, 분류유형코드, 단가, 1회투약량, 1일투약량, 총투여일수 또는 실시횟수, 금액, 일반명코드

요양기관 Table	요양기관대체키, 종별, 설립구분, 특수장비(CT,MRI,PET)유무, 시도구분, 병상수준, 50병상 당 의사·치과의사·한의사·간호사 수

Strengths and weaknesses

심평원의 환자표본자료는 방대한 원시데이터로부터 대표성을 확보할 수 있도록 추출한 자료로 기 구축된 자료를 활용할 수 있다는 점에서 비용과 시간을 아낄 수 있다[1]. 또한 충분한 타당성을 확보[2]하였으므로 모집단 추정에 효율적이다.

하지만, 전체환자표본자료를 활용 시 몇 가지 제한사항을 고려하여야 한다. 첫째로, 진단명의 정확성에 대한 문제를 고려하여야 한다. 진단명은 다빈도 경증 질환보다는 중증 질환이 더욱 정확성을 가지며, 외래보다는 입원이, 의원급보다는 병원급의 진단명 명 정확성이 더욱 높은 경향이 있다.[3] 따라서, 연구자의 조작적 정의를 통하여 진단명의 정확성을 적절히 고려하여 활용할 필요가 있다. 두 번째로는 특정 연령대나 희귀질환의 경우 추출되는 빈도가 낮기 때문에 설명력이 부족할 수 있다. 세 번째, 환자의 소득, 교육, 거주지, 몸무게, 키, 사망여부, 건강위험요인(흡연, 음주, 운동량) 등 수진자의 사회경제적요인 및 질환의 위험인자와 같은 환자특성이 부족하여 연구의 제한이 따른다. 이를 보완하기 위하여 타 기관과의 지속적인 접촉으로 자료 연계를 진행 중에 있다. 마지막으로, 환자표본자료는 cross sectional data 로 연도별로 동일한 환자의 추적 조사가 불가능하다. 개인정보보호의 이유로 수진자와 요양기관은 대체키로 가공되어 제공되고 있으며, 희귀질환, 법정전염병과 같은 민감한 질환의 경우 수진자의 개인정보가 식별될 수 있는 가능성이 커지므로 해당 질병 내역은 삭제되었다.

건강보험심사평가원은 보건의료관련 5 개 학회(대한예방의학회, 보건경제정책학회, 보건정보통계학회, 보건행정학회, 한국역학회)와 MOU 를 맺고 연구과제를 통한 환자표본자료의 타당도 평가를 수행하였으며, 주요 연구 결과 중 "우리나라 당뇨병

---

1 Kim JA, KimLY. Introduction and Usage of the National Health Insurance Claims Data.

2 Kim LY, Sakong J, Kim Y, Kim SR, Kim SK, Choei BH, et al. Developing the Inpatient Sample for the National Health Insurance Claims Data.

3 Park BJ, Seong JH, Park GD, Seo SW, Kim SH. Studying on improving diagnosis codes in National Health Insurance Claims Data.

유병률 추정 및 DPP-4 억제제 사용 양상 평가"에서 표본자료를 이용한 당뇨병 유병률 추정결과가 모집단 분석결과와 일치하였고, 혈당강하제 각 약효군별 처방률 추정결과가 모집단 분석결과와 일치하는 결과를 보였다. 또한 외래환자에서 각 약효군별 처방률은 모두 추정치의 95%신뢰구간 내에 참값 존재하였다.

"시력손실과 실명으로 인한 사회적 질병 부담비용 추계" 연구에서는 표본자료와 모집단 모두에서 여자가 남자에 비해 모든 주요안과질환(백내장, 녹내장, 황반변성, 당뇨망막변증, 망막정맥폐쇄)에서 의료이용 환자 비율이 높은 것으로 나타났으며 백내장, 녹내장, 환반변성의 연령별 추이는 모집단과 표본자료가 비슷한 양상을 가지는 것으로 나타났다.

Data Accessibility

전체환자표본자료의 신청 절차는 먼저 건강보험심사평가원 홈페이지를 접속하여 홈페이지상에서 환자표본자료 이용서약서의 제출과 함께 이루어 진다.

HIR 홈페이지→정부 3.0 정보공개→진료정보이용신청→표본자료<br>http://www.hira.or.kr/dummy.do?pgmid=HIRAA070001000312&cmsurl=/cms/open/02/01/02/index.html,<br><br>Tel: 02-2182-2601<br>Email: kshyun84@hiramail.net

환자표본 자료는 심의절차 없이 일정의 수수료를 지불한 후 환자표본자료를 직접 구매해서 사용할 수 있다. 자료형태는 DVD(text file 형식)로 제공되고 있다.
